# Long-term comparison of the efficacy of manual versus powered tooth brushing in adolescent orthodontic patients: a single-centre, parallel design randomized clinical trial

**DOI:** 10.1093/ejo/cjad042

**Published:** 2023-09-14

**Authors:** Ama Johal, Muftah Shagmani, Omar Alfuraih, Ian Arad

**Affiliations:** Department of Oral Bioengineering, Institute of Dentistry, Queen Mary University of London, Turner Street, Whitechapel, London E1 2AD, UK; Department of Oral Bioengineering, Institute of Dentistry, Queen Mary University of London, Turner Street, Whitechapel, London E1 2AD, UK; Department of Oral Bioengineering, Institute of Dentistry, Queen Mary University of London, Turner Street, Whitechapel, London E1 2AD, UK; Department of Oral Bioengineering, Institute of Dentistry, Queen Mary University of London, Turner Street, Whitechapel, London E1 2AD, UK

**Keywords:** tooth brush, manual, powered, gingival, plaque, index

## Abstract

**Background and objectives:**

To-date, there is no evidence comparing the long-term efficacy of powered and manual toothbrushes in adolescents undergoing fixed appliance treatment. The trial compared the efficacy of manual versus powered toothbrush in controlling plaque and gingival health in patients undergoing fixed treatment in respect of both the short- and long-term.

**Trial design:**

This was a randomized, parallel, controlled single-blind clinical trial, undertaken in a hospital setting, for which the consolidated standards of reporting trials guidelines were followed.

**Methods:**

Ninety-two adolescent participants planned to undergo fixed appliance therapy, were randomly assigned to either a manual or powered toothbrush, with allocation concealment. The outcome measures were plaque and gingival indices and bleeding on probing, assessed at baseline (prior to fixed appliance), one-, six- and 12-months.

**Results:**

The final sample included 84 participants, aged 12-18 (M=14.1, SD=1.93) years, with 40 (47%) were using a manual and 44 (52%) a powered toothbrush. The intervention (powered vs. manual toothbrush) itself appeared insignificant with regards to the gingival index (GI) (95%CI −0.1 – 0.03; *P*=0.26), plaque index (PI) (95%CI −0.13 – 0.14; *P*=0.93) and bleeding on probing (BoP) (95%CI −0.03 – 0.03; *P*=0.98) at any of the time points assessed. However, periodontal health indicators and plaque control significantly worsened (p<0.01), over the 12-month follow-up period, following placement of the fixed appliances placement.

**Conclusion:**

Whilst no differences were found between manual and powered toothbrushes in controlling plaque and gingival health, in participants undergoing fixed orthodontic treatment, both were suboptimal and highlighted the need for greater patient support and monitoring.

**Trial registration details:**

https://doi.org/10.1186/ISRCTN74268923  **Trial funding:** Colgate-Palmolive (USA)

## Introduction

Bacteria present in dental plaque are the primary aetiological agents in caries and periodontal disease. Therefore, effective tooth brushing remains an essential health practice to minimize plaque accumulation. Patients undergoing orthodontic treatment have greater difficulty in maintaining optimal oral hygiene levels, when compared with non-orthodontic patients, due to the presence of the appliances, increasing their susceptibility to gingivitis and enamel demineralization [1–4]. Orthodontic treatment with fixed appliances has also been strongly linked to gingival inflammation, bleeding, and increased pocket depth [5, 6]; however, there is no strong supporting evidence to suggest that it leads to irreversible attachment loss and periodontitis [7].

The meta-analysis undertaken by Kaklamanos and Kalfas [8] and a Cochrane review, presented a low–moderate level of evidence, with high levels of heterogeneity that powered toothbrushes perform better than manual toothbrushes in reducing plaque and gingivitis [9]. However, the majority of the included studies were on non-orthodontic patients (*n* = 44), and those studies which included orthodontic patients (*n* = 7) were of very limited duration (≤6 weeks). The Cochrane review suggests that further trials are needed, over a longer duration and with greater standardization of the methodology used [9]. A study carried out by Saruttichart *et al*. [10] investigated the effectiveness of a motionless ultrasonic toothbrush to a manual toothbrush in reducing dental plaque and gingival inflammation in a sample of 25 orthodontic patients. This was a single-blind randomized controlled trial, with a crossover design and intervening 30-day washout period. The study concluded that the manual toothbrush was ‘superior’ to the powered toothbrush, in reducing plaque around the fixed brackets in orthodontic patients, with no difference observed in terms of gingival health. However, the sample size was relatively small, and perhaps more importantly, the trial was again of a short (30 days) duration only.

Thus, to date, there is limited evidence comparing the efficacy of manual against powered toothbrushes in the short term (≤8 weeks) and importantly, no evidence comparing their efficacy in the long term in fixed appliance orthodontic patients.

This study aims to compare the efficacy of a powered versus manual toothbrush in controlling plaque and gingival health in participants undergoing fixed orthodontic treatment in respect of both the short term and long term.

## Materials and methods

### Participants, eligibility criteria, and setting

Ethical approval for this single-centre, hospital-based trial was granted by the National Research Ethics Services Committee (REC reference number: 14/LO/0003) and the trial registered (26.06.2014) with International Standard Randomised Controlled Trials Number (https://doi.org/10.1186/ISRCTN74268923).

The study population was drawn from those due to receive comprehensive orthodontic treatment, with a pre-adjusted edgewise fixed appliances, who fulfilled the following inclusion criteria:

1. Aged 12–18 years at the start of treatment
2. Brushed at least twice a day
3. Good general health and a non-smoker

Participants were excluded for the following reasons:

1. They already used a powered toothbrush
2. Special needs and learning difficulties
3. A history of periodontitis/attachment loss
4. Oral prophylaxis in the previous 4 weeks
5. Use of antibacterial mouth rinses or antibiotic therapy within the past month
6. The presence of five or more carious lesions requiring immediate restorative treatment.

### Trial design and setting

This was a randomized, parallel, controlled single-blind clinical trial, undertaken in a hospital setting, for which the consolidated standards of reporting trials (CONSORT) guidelines were followed [11]. All participants were recruited from those referred to the Hospital by their general dental practitioners and following a full orthodontic assessment, deemed to benefit from undergoing comprehensive fixed appliance therapy in both arches. Thus, all participants were approached before the commencement of treatment.

### Randomization, allocation concealment, and blinding

**Figure 1.**
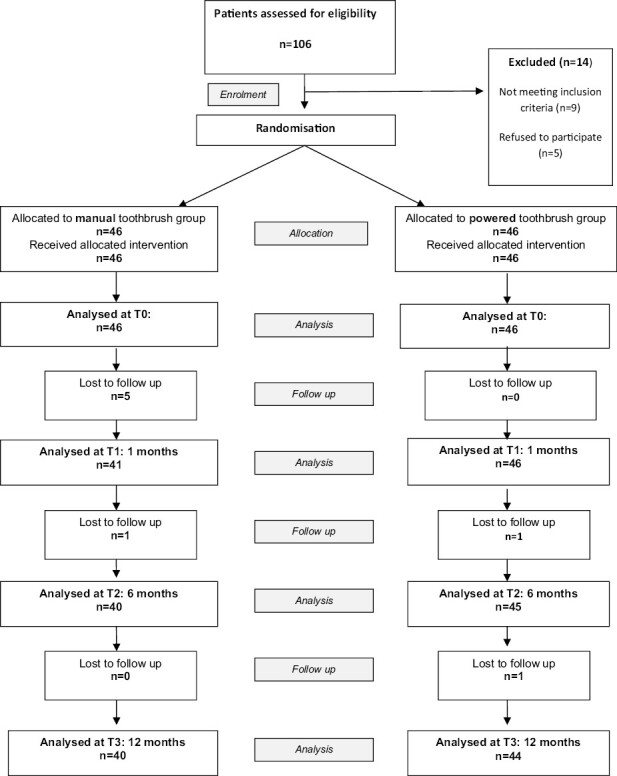
CONSORT flow diagram showing participant flow through the trial.

Participants who fulfilled the above selection criteria were identified and invited to take part in this study. Written informed consent and assent were obtained from the parent/guardian and participant, respectively. They were subsequently randomized (46 in each group) into either the intervention test (powered toothbrush) or control (manual toothbrush) groups (Fig. 1).

An electronic randomization programme was used, whereby an equal allocation sequence was generated for the 92 participants meeting the initial acceptance criteria. The numbers were randomly assigned to either one of the two groups and placed in pre-prepared opaque envelopes, ensuring they were all identical and tamper evident. In the presence of a research nurse, a single envelope was then selected randomly by the participants and opened immediately following the placement of their appliances.

The clinician assessing the outcomes was blinded to the randomization allocation, with a research nurse working independently to provide the oral hygiene instruction and to address any toothbrush or toothpaste replacement issues. Further blinding was undertaken during statistical analysis, aimed to further minimize bias.

### Interventions

Following appliance placement, the research nurse provided participants with either an Omron-powered sonic toothbrush (PT) and Triple Clean head (ProClinical A1500, Omron Corporation, Kyoto, Japan) or an Oral B Indicator Medium 35 manual toothbrush (MT; Proctor and Gamble, USA). The powered toothbrush heads and manual toothbrushes were replaced every 3 months, in line with the study protocol and as recommended by the ADA Council of Scientific Affairs [12]. The research nurse provided the participants with toothbrush instructions and in particular, for the powered toothbrush, the importance of ensuring charge was maintained, replacement of the heads and a chairside demonstration of the components, charging, and use on a typodont model.

All participants were provided Colgate cavity protection (Sodium Monofluorophosphate 1000 ppmF^-^, Sodium Fluoride 450 ppmF^-^; Colgate-Palmolive, USA) toothpaste and oral hygiene instructions, in line with the department’s policy, after fitting the fixed orthodontic appliances. At the commencement of the trial, participants were instructed to brush their teeth twice daily (in the morning and at bedtime) for a minimum of 2 min, they were encouraged to use an orthodontic interspace brush (Ortho-Care, England. UK) to optimize cleaning around the fixed appliances and demonstrated using a typodont model. During treatment, the clinician was responsible for monitoring the standard of oral hygiene and advising the patient and parent accordingly and, where necessary, reinforcing oral hygiene measures.

### Outcome measurements

Plaque and gingival scores assessed at baseline, before fixed appliance placement (T0), 1 month (T1), 6 months (T2), and 1 year (T3) along with an oral soft and hard tissue safety assessment. Data were collected by two trained and calibrated investigators (M.S. & O.A.) in the use of plaque and gingival indices.

**Figure 2.**
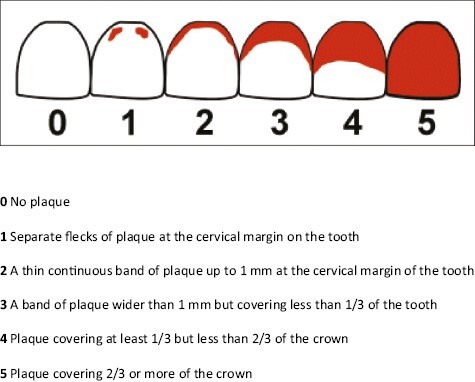
The Turesky modification of the Quigley–Hein plaque index. **0** No plaque. **1** Separate flecks of plaque at the cervical margin on the tooth. **2** A thin continuous band of plaque up to 1 mm at the cervical margin of the tooth. **3** A band of plaque wider than 1 mm but covering less than 1/3 of the tooth. **4** Plaque covering at least 1/3 but less than 2/3 of the crown. **5** Plaque covering 2/3 or more of the crown.

Plaque index (PI), gingival index (GI), and bleeding on probing (BoP) were recorded, at six sites per tooth, three on the buccal (mesio-buccal, mid-buccal, and disto-buccal), and three on the lingual tooth surfaces (mesio-lingual, mid-lingual, and disto-lingual) of the permanent dentition. Plaque scores were assessed using the Turesky modification of the Quigley–Hein plaque index (Fig. 2) [13–15]. Gingival health scores were assessed using both the Löe and Silness gingival index [15, 16] (Table 1) and bleeding on probing, in order to quantify the number of sites affected, as recommended by Robinson *et al*. [17] and the American Dental Association (ADA) [18].

**Table 1.** The Löe and Silness gingival index.

Score	Criteria
0	No inflammation
1	Mild inflammation, slight change in colour, slight oedema, no bleeding on probing
2	Moderate inflammation, moderate glazing, redness, bleeding on probing.
3	Severe inflammation, marked redness and hypertrophy, ulceration, tendency to spontaneous bleeding

Interpretation: severe (scores between 2.1 and 3.0), moderate (scores between 1.1 and 2.0), mild (score between 0.1 and 1.0), no inflammation (score of <0.1).

The soft tissues (tongue, hard and soft palate, gingivae, muco-buccal folds, buccal mucosa, and floor of mouth) and hard tissues (orthodontic appliance attachments and the cervical portions of the teeth) were all assessed for trauma and or damage.

### Statistical analysis

#### Sample size calculation

According to the trial carried out by Clerehugh *et al*. [19], *a priori* power calculation was undertaken to detect a difference in full mouth plaque score reduction of 0.25, with an observed variance of 0.3, applying two-sided testing with an α of 0.05 and β of 0.10. This was in line with the American Dental Association’s ‘Acceptance Programme Guidelines for Toothbrushes’ [18]. Thus, a total sample size of 80 participants was estimated. Based on previous studies, which accounted for their losses, a total sample size of 92 was to be recruited, thus allowing for 12 drop-outs in this current trial [19,20].

#### Statistical methods

Data analysis was carried out using JMP®, Version 14 Pro (SAS Institute, Inc., Cary, NC, 1989-2019) software. The data (PI, GI, and BoP) were tested for normality and an intention-to-treat analysis was applied. The three primary outcomes (plaque scores, gingival index, and bleeding on probing) were collected per tooth, at each time point. The data were structured such that the unit of analysis was the specific tooth ‘nested’ within the participant’s mouth, repeated over time (1, 6, and 12 months). This approach allowed the outcomes per individual tooth to be monitored in the mouth, permitting a more detailed analysis rather than averaging measurements per person which would mask upfront the majority of the variance. The interactions were tested using parameter estimates. The details of the models applied are described below and included coefficients per each comparison. Therefore, a mixed-effects (fixed and random) multiple linear regression approach was chosen. Using the random coefficients, this approach allowed for an elaborate error framework in accordance with the nested structure of the data without violating the independency assumption, using the Restricted Maximum likelihood (REML) approach instead of the classical repeated measures structure, the model was capable of dealing with any missing observations (when individual teeth were not present e.g. second molars) while balancing their weight with respect to the variance. In these models, time point (1-, 6-, and 12-month time points) and tooth were regarded as within participant effects, while toothbrush was the only between participant effect. Applying the full factorial mixed-effects multiple linear regression strategy for each independent variable (at the tooth level). The model estimated included the following fixed effects: Toothbrush type, Time point, Tooth, Toothbrush × Time point, Toothbrush × Tooth, Time point × Tooth, Toothbrush × Time point × Tooth. In addition, the models included the following random effects: Subject No., Subject No. × Time point, and Subject No × Tooth; each nested within toothbrush type. This allows the estimation of each individual tooth as well as all of them together at the patient level. The data were analysed on an intention-to-treat basis, with all participants included, according to their original allocation, regardless of the outcome of treatment.

The reproducibility of measurements for the two investigators was assessed using the interclass correlation coefficient. For all analyses, a *P*-value of <0.05 was regarded as significant.

#### Examiner alignment and assessment

During examiner alignment, both examiners (M.S. and O.A.) and a research nurse (S.S.) were instructed and coached in the proper use of the outcome measurements (GI, BoP, and PI), before participant recruitment to the trial [21]. This was undertaken by an experienced periodontist using a power-point presentation, models, and discussion of the challenges.

A clinical assessment study to assess intra-examiner reproducibility of the measurements, was performed on 20 randomly selected participants. PI, GI, and BoP were recorded, at six sites per tooth, (described above), at the same visit with a 30-min interval. A weighted concordance Kappa’s index was performed to assess agreement between repeated measurements. Concordance of measurements was found to be good for GI (0.758, 95% CI 0.739–0.778) and BoP (0.77, 95% CI 0.753–0.796) and very good for PI (0.845, 95% CI 0.829–0.862).

## Results

### Participant flow

Recruitment commenced in July 2014 and was completed in November 2017 (a 3-year 4-month rolling recruitment period). The recruitment and follow-up of all participants can be seen in the CONSORT flow diagram (Fig. 1). Of the 106 participants consecutively assessed for eligibility, 14 were excluded from the study, as 9 did not meet the inclusion criteria and a further 5 declined to take part in the trial. Thus, 92 participants were randomized at baseline (T0) to receive either an MT or PT. There were eight drop-outs over the 12-month follow-up period, three participants failed to attend for assessment, and five disliked the toothbrush that they had been given at T0 and were subsequently excluded from the study (Fig. 1). At 6-month (T2) follow-up, there were 40 and 45 participants in the control and intervention groups, respectively and at the 12-month follow-up, there was 1 further drop-out, with the participant failing to attend in the intervention group.

### Baseline data

The final sample included 92 participants in the ages of 12–18 years (*M* = 14.7, SD = 2.3), of which 46 were using an MT and 46 a PT. This accumulates to 7,008 individual tooth measurements. Table 2 shows the baseline demographics; mean age, gender distribution, and incisor classification of the participants were not significantly different between groups following the group randomization (*P* > 0.05). Table 3 reports the descriptive statistics for GI, PI, and BoP for both MT and PT groups at each time point (T0–T3).

**Table 2.** Baseline demographic and clinical characteristics of the sample (*n* = 92).

	Overall sample (*n* = 92)	Manual toothbrush (*n* = 46)	Powered toothbrush (*n* = 46)
Age (mean, SD)	14.07 (2.3)	13.93 (2.82)	14.21 (1.78)
Gender			
Male	48 (52%)	28 (60%)	20 (43%)
Female	44 (48%)	18 (40%)	26 (57%)
Incisor classification			
Class I	13 (14.1%)	6 (13.0%)	7 (15.2%)
Class II Division 1	46 (50.0%)	24 (52.2%)	22 (47.8%)
Class II Division 2	11 (12.0%)	4 (8.7%)	6 (13.0%)
Class III	22 (23.9%)	13 (28.3%)	9 (19.6%)

**Table 3.** Descriptive statistics for gingival, bleeding on probing, and plaque indices for manual and power toothbrushes groups at each time point.

		Gingival Index	Bleeding on probing	Plaque Index
Time	Toothbrush	Mean	Mean	Mean
Baseline	Manual	1.03	0.23	1.86
	Power	1.08	0.26	1.78
1 month	Manual	1.08	0.21	2.35
	Power	1.16	0.24	2.36
6 months	Manual	1.01	0.21	2.45
	Power	1.11	0.19	2.54
12 months	Manual	1.02	0.19	2.62
	Power	1.00	0.11	2.40

### Numbers analysed for each outcome

For the MT group, a total of 46, 41, 40, and 40 participant’s data were analysed at T0, T1, T2, and T3, respectively (Fig. 1). For the PT group, a total of 46, 46, 45, and 44 participant’s data were analysed at T0, T1, T2, and T3, respectively.

### Changes in outcome measures

**Figure 3.**
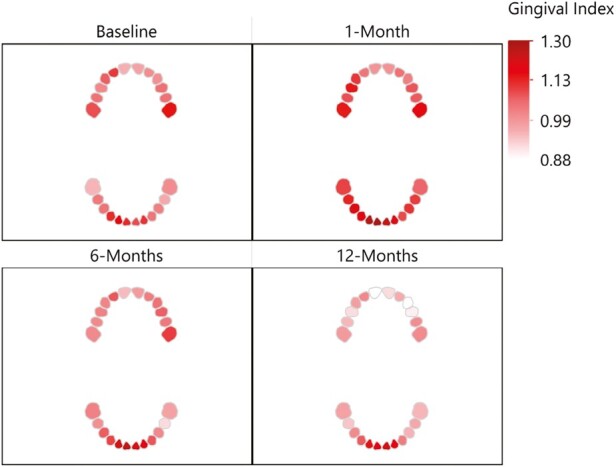
The interaction observed between the specific tooth in the participant’s mouth and gingival index scores.

**Figure 4.**
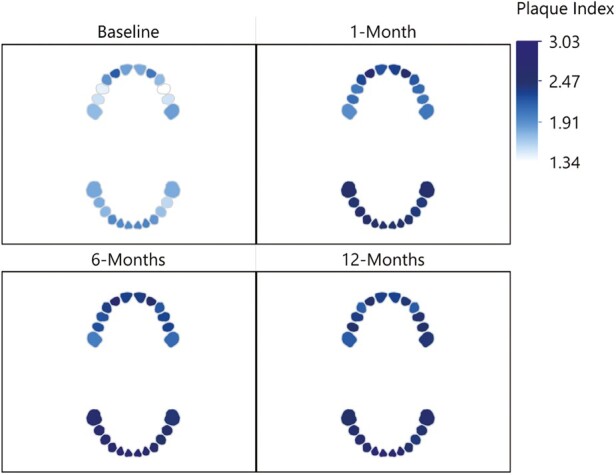
The interaction observed between the specific tooth in the participant’s mouth and plaque index scores.

**Figure 5.**
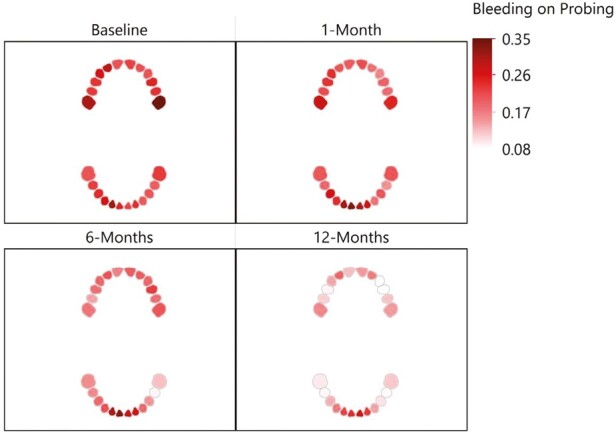
The interaction observed between the specific tooth in the participant’s mouth and bleeding on probing scores.

**Figure 6.**
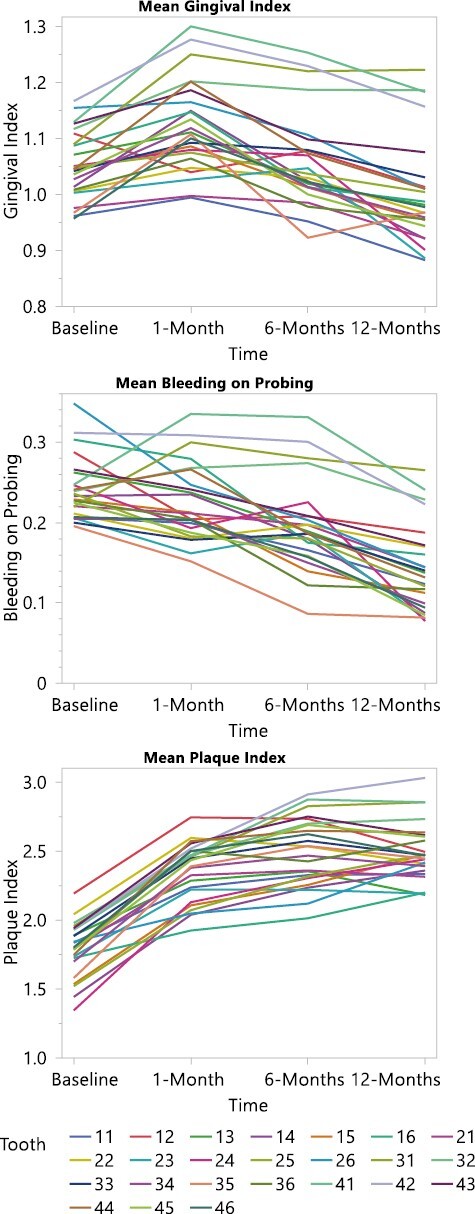
Spaghetti plots for mean Gingival Index, bleeding on probing, and Plaque Index to show changes over time and per tooth in the mouth.

The following section presents a separate analysis for each dependent variable: GI, PI and BoP from baseline (T0; immediately before placement of the fixed appliance) to 12-months (T3; Tables 3–6). The interaction observed between the specific tooth in the participant’s mouth and these dependent variables over the study follow-up time points (T0–T3) are illustrated in Figs 3–5. Here, a notable and characteristic pattern of observation was seen for GI and BoP, with an increased score for these variables observed at 1 month, which reduced in relation to both dental arches over the subsequent follow-up period, with the exception of the lower incisor and posterior molar regions, where the scores remained high. For PI, the initial increase was followed by further deterioration and higher levels of plaque being recorded at each subsequent time point. Furthermore, Fig. 6 presents a spaghetti graph for GI, PI, and BoP, in which the mean value for each tooth was connected over time. The intersecting and overlapping patterns revealed that the data was rich and complex, indicating a need for a model that decomposed the variance by tooth, person, and time to better represent the dependency structure of the observations.

**Table 4.** Mixed-effects multiple linear regression for Gingival Index.

Gingival index			95% CI	
Parameter Estimates - Fixed Effects	Estimate	Prob>|t|		
Intercept	1.05	<0.0001	0.99	1.11
Toothbrush [Manual]	−0.03	0.263	−0.1	0.03
Time [Baseline]	−0.01	0.715	−0.06	0.04
Time [1 month]	0.07	0.016	0.01	0.12
Time [6 months]	−0.01	0.765	−0.06	0.05
Toothbrush [Manual] × Time [Baseline]	−0.02	0.489	−0.07	0.04
Toothbrush [Manual] × Time [1 month]	−0.01	0.850	−0.06	0.05
Toothbrush [Manual] × Time [6 months]	−0.02	0.568	−0.07	0.04

*R* ^2^ = 0.69, *R*^2^ Adj = 0.68, *N* = 7,008.

Model includes fixed effects for tooth and its interaction with time and toothbrush.

**Table 5.** Mixed-effects multiple linear regression for bleeding on probing.

Parameter estimates—Fixed Effects	Estimate	Prob>|t|	95% CI	
Intercept	0.19	<0.0001	0.16	0.23
Toothbrush [Manual]	0.003	0.9845	−0.03	0.03
Time [Baseline]	0.03	0.0225	0.005	0.06
Time [1 month]	0.03	0.0554	−0.001	0.06
Time [6 months]	−0.01	0.5049	−0.04	0.02
Toothbrush [Manual] × Time [Baseline]	−0.02	0.1503	−0.05	0.01
Toothbrush [Manual] × Time [1 month]	−0.02	0.2541	−0.05	0.01
Toothbrush [Manual] × Time [6 months]	0.004	0.799	−0.02	0.03

*R* ^2^ = 0.62, *R*^2^ Adj = 0.61, *N* = 7,007

Model includes fixed effects for tooth.

**Table 6.** Mixed-effects multiple linear regression for Plaque index.

Parameter estimates—Fixed effects	Estimate	Prob>|t|	95% CI	
Intercept	2.28	<0.0001	2.15	2.41
Toothbrush [Manual]	0.01	0.9252	−0.13	0.14
Time [Baseline]	−0.44	<0.0001	−0.55	−0.34
Time [1 month]	0.09	0.0958	−0.02	0.19
Time [6 months]	0.15	0.0058	0.04	0.25
Toothbrush [Manual] × Time [Baseline]	0.01	0.7903	−0.09	0.12
Toothbrush [Manual] × Time [1 month]	−0.0001	0.9985	−0.1	0.1
Toothbrush [Manual] × Time [6 months]	−0.11	0.0339	−0.22	−0.01

*R* ^2^ = 0.67, *R*^2^ Adj = 0.66, *N* = 7,008

Model includes fixed effects for tooth.

#### Gingival index

The mixed-effects multiple linear regression was used to estimate the difference in GI scores across all participant teeth (Table 4). The intervention (PT vs. MT) main effect appeared to be insignificant (*B* = −0.03, *P* = 0.26; 95% CI −0.1 to 0.03). In addition, the interaction of time point (T0–T3) and toothbrush was insignificant at all time points. Yet, time point by itself had a significant main effect. Such that, at T1 the index was significantly higher than the overall average (*B* = 0.07, *P* = 0.016; 95% CI 0.01–0.12).

#### Bleeding on probing

Table 5 provides the parameter estimates for the mixed-effects multiple linear regression for BoP. Here, the effect of the toothbrush type (PT vs. MT) was again found to be insignificant (*B* = 0.003, *P* = 0.984; 95% CI −0.03 to 0.03). Furthermore, none of the interaction terms of time and toothbrush were significant. However, a significant time effect was observed. At baseline, the reading was significantly higher than the overall recorded average score (*B* = 0.03, *P* = 0.02; 95% CI 0.005–0.06).

#### Plaque Index

In the mixed effects model for PI (Table 6), similar to GI and BoP, the main fixed effect of the toothbrush type (PT vs. MT) was again found to be insignificant (*B* = 0.01, *P* = 0.93; 95% CI −0.13 to 0.14). In addition, all three parameters for toothbrush interaction with time were found to be insignificant. Otherwise, the effect of time was found to be significant. At baseline, the reading was significantly lower (*B* = −0.44, *P* < 0.001; 95% CI −0.55 to −0.34), while at 6 months, it was significantly higher than overall (*B* = 0.15, *P* = 0.006; 95% CI 0.04–0.25).

### Harms

All participants underwent a comprehensive evaluation of their intra-oral hard and soft tissues at baseline and follow-up. At the 12-month (T3) follow-up period, there was no harm reported or observed as a result of either intervention, confirming their safety and tolerance. Furthermore, no participant was removed from the trial on the basis that their oral hygiene proved to be unsatisfactory, requiring their fixed orthodontic appliances to be removed.

## Discussion

To date, no long-term RCT designed to evaluate the impact of MT versus PT in an orthodontic population has been reported, while more recent studies have once again only reported short-term (<8 weeks) evaluations [22].

A key requirement of any participant undergoing fixed appliance therapy is the need to maintain optimal oral hygiene. Furthermore, the long-term nature of orthodontic treatment increases the likelihood of both hard (enamel demineralization, with white spot lesions or caries) and soft (gingivitis, hyperplasia, and periodontal pocketing/attachment loss) tissue complications, in the absence of this optimal cleaning. Thus regular tooth brushing becomes essential, and with the availability of PT and their inherent appeal, there is a need for robust clinical evidence of their effectiveness over both the short term but perhaps more importantly, the long term [9,23]. This study is the first to adopt a prospective longitudinal randomized clinical trial design, in line with the CONSORT guidelines [11], in order to address the significant shortcomings in the current literature. Despite the conclusions of a Cochrane systematic review of manual versus powered toothbrushes, reporting the latter to be more effective in the reduction of plaque and gingivitis, in both the short term and long term [9], the authors reported a number of important limitations in the review. These primarily related to the fact that only 7 (14%) of the 51 studies included orthodontic participants, all of which were short term (≤6 weeks) in their follow-up duration and highlighted significant variability in their methodology, resulting in high levels of heterogeneity and only one study was assessed as being at low risk of bias [9]. On closer inspection, three of these studies found no differences in the PI and GI [24–26] two studies found that powered toothbrushes contributed to lower plaque and gingival scores [20,27] and the remaining two studies found a significant reduction in gingivitis only [19,28]. It should also be noted that one of these studies remains unpublished [28].

A further strength of the current trial was that following the initial protocol registration with the International Standard Randomised Controlled Trials Centre, no protocol deviations were reported, thus minimizing the risk of bias, through e.g. selective reporting. Furthermore, the trial sample retained its power, with appropriate allowance being made for potential drop-outs, with the observed rate being consistent with previous studies in the same field [19,20]. The final sample within each group was both reflective in the age of participants undergoing fixed appliance therapy in adolescence and was evenly distributed by gender, thus minimizing any potential confounders. As identified above, the present trial followed the recommendations of the Cochrane review, and aimed to limit study heterogeneity by utilizing the recommended outcome measures [9].

The present trial could detect no difference in three primary outcomes (GI, PI, and BoP), at any of the time points from 1 to 12 months, in participants undergoing comprehensive fixed appliance therapy in both arches, in terms of the toothbrush type. These findings do not support the superiority of a specific PT in maintaining optimal health when compared with an MT for those wearing orthodontic appliances. The findings are in contrast with a number of previous studies, which as highlighted above, are at risk of bias, with significant limitations, and perhaps most importantly, fail to take into account the long-term nature of orthodontic treatment. In a recent trial, designed to quantify the duration of tooth brushing, with MT and PT, in children with fixed appliances, no differences were detected in their effectiveness, with a reported brushing time of approximately 3 min [29]. Furthermore, the authors reported that despite the introduction of a disclosing agent to determine the duration of cleaning, the children could only achieve an effective plaque reduction of 76% [29]. Perhaps of greater significance is that the current trial highlights the fact that the standard of oral hygiene in adolescent patients, irrespective of the toothbrush type, remains suboptimal and consequently increases the risk of detrimental harm as a consequence of treatment. Thus, there appears a need for orthodontists to offer their adolescent patients a behaviour management program to optimize periodontal health. While there is evidence of the reported benefit of such an intervention in adults with mild to moderate periodontitis, the authors highlighted the fact that to bring about a meaningful change in lifestyle would require repeated engagement over time [30]. This model particularly lends itself to orthodontic treatment as patients are seen on a 4–6 weekly basis throughout their orthodontic treatment.

This study did find a significant interaction between the toothbrush type, the follow-up period, and specific tooth in the participant’s mouth. Specifically, an increase was observed in the GI, PI, and BoP at 1 month, which was reduced during the subsequent follow-up periods, with the exception of the lower incisor and posterior molar regions, where the scores remained high. The latter reflects well in terms of the more recently described concept of some bleeding being commensurate with periodontal health [31]. The literature has previously identified the risk of gingival inflammation, within a month of placement of fixed appliances [1, 2]. However, more recently, Erbe *et al*. [32] described in a similar format to the current study, the mouth, and teeth into focus care areas. The authors reported the same three regions (right and left posterior and lower labial) as being most susceptible to plaque accumulation. Importantly, the literature has already drawn attention to the fact that, in the absence of orthodontic appliances, relatively inaccessible areas of the dentition to the toothbrush remain at the greatest risk of hard and soft tissue damage [33–35]. This naturally assumes a greater level of importance if we accept the presence of fixed appliances result in a greater plaque retention capability, and the principle target population are adolescents who may not have the awareness or motivation for the need to maintain optimal oral hygiene [36]. It is therefore important to offer support and encouragement to these adolescent patients, with careful follow-up monitoring required, with regular dental care being provided in addition to the planned orthodontic care. The concept of a dental map or focus care area, in turn could also serve to help both clinicians and patients to visualize the areas of the dentition that typically require more attention and, as such, are at risk. This, in turn, may facilitate a better dialogue between the patient, parent/guardian, and clinician and ultimately collaboration towards an optimal result [32].

While every effort was made in the design and execution of the current randomized clinical trial to address the many shortcomings in the present literature, nevertheless, there are inherent limitations. The trial was based in a single-hospital setting, and therefore, its findings may not be generalizable. The study was not double-blind, as participants were clearly aware of the choice of tooth brush to be used. However, the operator and outcome assessment, along with the data analysis, were all performed blind.

The specific powered toothbrush selected for the present study used a sonic triple clean head, and alternatives are now available, which may be more effective [37]. Indeed, there have been continuous advancements in powered toothbrush technology since the start of this study in 2015, with the inclusion of pressure sensors, inbuilt timers and inbuilt timers and particularly in the area of ‘connected’ power toothbrushes which employ tracking capabilities to personalize real-time guidance during brushing and which enable users to monitor their brushing behaviours. There is growing evidence that the inclusion of these interactive technologies can enhance compliance with powered toothbrushes, resulting in detectable differences in the outcome scores [32].

## Conclusions

No differences have been found between a manual and sonic-powered toothbrush in controlling plaque and gingival health in participants undergoing fixed orthodontic treatment in either the short term or long term.

## Data Availability

The data underlying this article will be shared on reasonable request to the corresponding author.

## References

[1] Zachrisson BU, Zachrisson S. Caries incidence and orthodontic treatment with fixed appliances. Scand J Dent Res 1971;79:183–92. 10.1111/j.1600-0722.1971.tb02008.x 5285724

[2] Zachrisson S, Zachrisson BU. Gingival condition associated with orthodontic treatment. Angle Orthod 1972;42:26–34. 10.1043/0003-3219(1972)042<0026:GCAWOT>2.0.CO;2 4500561

[3] Atack NE, Sandy JR, Addy M. Periodontal and microbiological changes associated with the placement of orthodontic appliances. A review. J Periodontol 1966;67:78–85. 10.1902/jop.1996.67.2.78 8667140

[4] Farronato G, Giannini L, Galbiati G et al. Oral tissues and orthodontic treatment: common side effects. Minerva Stomatol 2013;62:431–46. 24270203

[5] Sallum EJ, Nouer DF, Klein MI et al. Clinical and microbiologic changes after removal of orthodontic appliances. Am J Orthod Dentofacial Orthop 2004;126:363–6. 10.1016/j.ajodo.2004.04.017 15356501

[6] Naranjo AA, Triviño ML, Jaramillo A et al. Changes in the subgingival microbiota and periodontal parameters before and 3 months after bracket placement. Am J Orthod Dentofacial Orthop 2006;130:275.e17–22. 10.1016/j.ajodo.2005.10.022 16979483

[7] Papageorgiou SN, Papadelli AA, Eliades T. Effect of orthodontic treatment on periodontal clinical attachment: a systematic review and meta-analysis. Eur J Orthod 2018;40:176–94. 10.1093/ejo/cjx052 29106513

[8] Kaklamanos EG, Kalfas S. Meta-analysis on the effectiveness of powered toothbrushes for orthodontic patients. Am J Orthod Dentofacial Orthop 2008;133:187.e1–187.e14. 10.1016/j.ajodo.2007.07.015 18249278

[9] Yaacob M, Worthington HV, Deacon SA et al. Powered versus manual toothbrushing for oral health. Cochrane Database Syst Rev 2014;6:CD002281. 10.1002/14651858.CD002281.pub3 PMC7133541 24934383

[10] Saruttichart T, Chantarawaratit P, Leevailoj C et al. Effectiveness of a motionless ultrasonic toothbrush in reducing plaque and gingival inflammation in patients with fixed orthodontic appliances. Angle Orthod 2016;87:279–85. 27636178 10.2319/042516-334.1 PMC8384361

[11] Schulz KF, Altman DG, Moher M. CONSORT 2010 statement: updated guidelines for reporting parallel group randomized trials. BMJ 2010;340:c332–338. 20332509 10.1136/bmj.c332 PMC2844940

[12] American Dental Association Council on Scientific Affairs. (2011). Toothbrush care: Cleaning, Sorting and replacement. Available at: http://www.ada.org/en/about-the-ada/ada-positions-policies-and-statements/statement-on-toothbrush-care-cleaning-storage-and- [Accessed August 2020].

[13] Quigley GA, Hein JW. Comparative cleaning efficiency of manual and power brushing. J Am Dent Assoc 1962;65:26–9. 14489483 10.14219/jada.archive.1962.0184

[14] Turesky SS. What is the role of dental calculus in the etiology and progression of periodontal disease? (1972). J Periodontol 41:285–86. 5267746

[15] Löe H, Silness J. Periodontal disease in pregnancy prevalence and severity. Acta Odontologica Scandanavian 1963;21:522–51. 10.3109/00016356309011240 14121956

[16] Löe H. The Gingival Index, the Plaque Index and the Retention Index systems. J Periodontol 1967;38:610–6. 10.1902/jop.1967.38.6.610 5237684

[17] Robinson PG, Deacon SA, Deery C et al. Manual versus powered toothbrushing for oral health. Cochrane Database Syst Rev 2005;18:CD002281. 10.1002/14651858.CD002281.pub2 15846633

[18] American Dental Association Council on Scientific Affairs. (2012). Acceptance Program Guidelines for Toothbrushes. Available at: http://www.ada.org/~/media/ADA/Science%20and%20Research/Files/guide_toothbrushes.ashx [Accessed 13 December 2014].

[19] Clerehugh V, Williams P, Shaw WC et al. Practice-based randomised controlled trial of the efficacy of an electric and a manual toothbrush on gingival health in patients with fixed orthodontic appliances. J Dent 1988;6:633–39. 10.1016/s0300-5712(97)00065-1 9793284

[20] Ho HP, Niederman R. Effectiveness of the sonicare sonic toothbrush on reduction of plaque, gingivitis, probing pocket depth and subgingival bacteria in adolescent orthodontic patients. J Clin Dent 1997;8:15–9. 9487840

[21] Hefti AF, Preshaw PM. Examiner alignment and assessment in clinical periodontal research. Periodontology 2000 2012;59:41–60. 10.1111/j.1600-0757.2011.00436.x 22507059

[22] Elshehaby MM, Mofti B, Montasser MA et al. Powered vs. manual tooth brushing in patients with fixed orthodontic appliances: a systematic review and meta-analysis. Am J Orthod Dentofacial Orthop 2020;158:639–49. 10.1016/j.ajodo.2020.04.018 32951930

[23] Erbe C, Klees V, Braunbeck F et al. Comparative assessment of plaque removal and motivation between a manual and an interactive power toothbrush in adolescents with fixed orthodontic appliances: a single-center, examiner-blind randomized controlled trial. Am J Orthod Dentofacial Orthop 2019;155:462–72. 10.1016/j.ajodo.2018.12.013 30935601

[24] Pucher JJ, Lamendola-Sitenga K, Ferguson D et al. The effectiveness of an ionic toothbrush in the removal of dental plaque and reduction on gingivitis in orthodontic patients. J West Soc Periodontol Periodontal Abstr 1999;47:101–7. 11830911

[25] Hickman J, Millett DT, Sander L et al. Powered vs manual tooth brushing in fixed appliance patients: a short term randomized clinical trial. Angle Orthod 2002;72:135–40. 10.1043/0003-3219(2002)072<0135:PVMTBI>2.0.CO;2 11999936

[26] Costa MR, Silva VC, Miqui MN et al. Efficacy of ultrasonic, electric and manual toothbrushes in patients with fixed orthodontic appliances. Angle Orthod 2007;77:361–6. 10.2319/0003-3219(2007)077[0361:EOUEAM]2.0.CO;2 17319775

[27] Silvestrini Biavati A, Gastaldo L, Dessì M et al. Manual orthodontic vs. oscillating-rotating electric toothbrush in orthodontic patients: a randomised clinical trial. Eur J Paediatr Dent 2010;11:200–2. 21250772

[28] Singh A, Maddalozzo D, Geivelis M et al. A clinical comparison of the Butler GUM Pulse plaque remover and manual toothbrushing in adolescent orthodontic patients. Unpublished.

[29] Koretsi V, Kline R, Herreiner P et al. Duration of toothbrushing with fixed appliance: a randomized crossover clinical trial. Eur J Orthod 2022;44:252–7. 34849694 10.1093/ejo/cjab075

[30] Donos N, Suvan JE, Calciolari E et al. The effect of a behavioural management tool in adults with mild to moderate periodontitis. A single-blind, randomized controlled trial. J Periodontal Res 2021;56:46–57. 10.1111/jre.12790 32959898

[31] Lang NP, Bartold PM. Periodontal health. J Periodontol 2018;89:S9–S16. 10.1002/JPER.16-0517 29926938

[32] Erbe C, Ccahuana-Vasquez RA, Ferrari-Peron P et al. A comparative assessment of plaque removal and toothbrushing compliance between a manual and an interactive power toothbrush among adolescents: a single-center, single-blind randomized controlled trial. BMC Oral Health 2018;18:130–8. 30075780 10.1186/s12903-018-0588-1 PMC6091059

[33] Cancro LP, Fischman SL. The expected effect on oral health of dental plaque control through mechanical removal. Periodontology 2000;8:60–74. 10.1111/j.1600-0757.1995.tb00045.x 9567946

[34] Tanner AC, Kent R, van Dyke T et al. Clinical and other risk indicators for early periodontitis. J Periodontol 2005;76:573–81. 10.1902/jop.2005.76.4.573 15857098 PMC1224718

[35] Creeth JE, Gallagher A, Sowinski J et al. The effect of brushing time and dentifrice on dental plaque removal in vivo. J Dent Hyg 2009;83:111–6. 19723429

[36] Yildrim S, Kayaah-Yuksek S. The effects of motivational methods applied during toothbrushing on children’s oral hygiene and periodontal health. Paediatric Dentistry 2020;42:424–30. 33369552

[37] Büchel B, Reise M, Klukowska M et al. A 4-week clinical comparison of an oscillating-rotating power brush versus a marketed sonic brush in reducing dental plaque. Am J Dent 2014;27:56–60. 24902407

